# sGRP78 enhances selective autophagy of monomeric TLR4 to regulate myeloid cell death

**DOI:** 10.1038/s41419-022-05048-5

**Published:** 2022-07-07

**Authors:** Zhenghao Wu, Zhuoshuo Xu, Xiaoqi Zhou, Heli Li, Liang Zhao, Yibing Lv, Yanyan Guo, Guanxin Shen, Yong He, Ping Lei

**Affiliations:** 1 Department of Immunology, School of Basic Medicine, Tongji Medical College, Huazhong University of Science and Technology, 430030 Wuhan, China; 2 Department of Nuclear Medicine and PET Center, Zhongnan Hospital of Wuhan University, 430071 Wuhan, China; 3 Department of Breast and Thyroid Surgery, Union Hospital, Tongji Medical College, Huazhong University of Science and Technology, 430022 Wuhan, China

**Keywords:** Immune cell death, Innate immune cells, Signal transduction

## Abstract

Soluble glucose regulated protein 78 (sGRP78) has long been suggested as a mediator resolution of inflammation. We previously reported that sGRP78 induced the rapid endocytosis of TLR4 with defective TLR4 signaling. To elucidate the underlying mechanisms, in this study, we investigated how sGRP78 influenced the behavior and trafficking of TLR4 in myeloid cells. It was found that sGRP78 promoted LPS endocytosis with monomeric TLR4. This internalized monomeric TLR4 formed complexes with p62–LC3, and was degraded in autolysosomes. Furthermore, the sGRP78-enhanced autophagy-dependent TLR4 degradation caused apoptosis and ferroptosis in myeloid cells, contributing to the sGRP78-mediated resolution of inflammation. These reports establish innovative mechanisms for endotoxin clearance and immune regulation by TLR4 degradation, linking innate immunity with multiple ancient processes, including autophagy, apoptosis, and ferroptosis, together through a shared resolution-associated molecular pattern (RAMP)—sGRP78.

## Introduction

The 78-kDa glucose-regulated protein (GRP78), a member of the highly conserved heat shock 70 kDa protein (HSP70) family, is constitutively expressed in the endoplasmic reticulum (ER) [1]. GRP78 regulates the balance between cell viability and apoptosis by sustaining ER protein folding capacity and maintaining ER stresses in their inactive state [2]. Upregulation of GRP78 is induced by ER stress, leading to its cell surface expression and secretion into the extracellular compartment [3]. We have previously reported that secreted GRP78 (sGRP78) induced splenic B cells into regulatory IL-10^+^PD-L1^hi^FasL^hi^ B cells [4], as well as BMDCs into tolerogenic DCs with low MHC-II expression and high levels of B7-H3, B7-H4 [5]. In addition, sGRP78 inhibited the release of inflammatory cytokines, including IFNβ, IL1β, IL6, and TNFα in LPS-conditioned BMDCs and BMDMs [6]. For its potent immunomodulatory properties, sGRP78 has been defined as one of the resolution-associated molecular patterns (RAMPs) to counterbalance the inflammatory effects of pathogen-associated molecular patterns (PAMPs) and damage-associated molecular patterns (DAMPs) to maintain immune homeotosis [7].

When we explored how sGRP78 exerted such anti-inflammatory functions, we found that sGRP78 could inhibited TLR4 signaling through the promotion of TLR4 endocytosis and reduction of its expression on the surface of DCs [6]. As representative pattern recognition receptor (PRR), TLRs play an important role in recognizing PAMPs and DAMPs and consequently induce activities that stimulate immunity and host defense [8]. Cell surface TLR4 recognizes LPS, the typical outer membrane component of Gram-negative bacteria, and activates myddosome (MyD88)-dependent signaling in the plasma membrane [9]. Then TLR4 is selected as cargo for endocytosis to initiate intracellular triffosome (TRIF)-dependent pathway, including IRF3 phosphorylation and IFN-β release [6]. However, we found that sGRP78-induced TLR4 internalization was not accompanied by TLR4 endocytosis-related TRIF signaling. On the contrary, sGRP78-conditioned cells downregulated their TRIF-dependent IRF3 phosphorylation and IFNβ release [7]. Hence, it is worthy of our investigation why internalized TLR4 is inactive under the context of sGRP78.

Upon sGRP78 stimulation, internalized TLR4 was observed to co-localize with the late endosomal/lysosomal marker protein LAMP1, suggesting the degradation of TLR4 in lysosomes [6]. Proteins destined for lysosomal degradation can reach the lysosome by a variety of means and autophagy is one of them in mammalian cells. After the initial isolation membrane is grown, the formed autophagosome fuses with lysosomal vesicles and delivers the engulfed cargo for degradation. The autophagy-lysosomal pathway is a major mechanism for degrading intracellular macromolecules, having multiple effects on immunity. It controls inflammation through regulatory interactions with innate immune signaling pathways by removing endogenous inflammasome agonists and affecting the secretion of immune mediators [10]. LPS-induced TLR signaling activates autophagy to remove pathogens in autophagosomes [11, 12]. In addition, autophagy inhibits TLR4-dependent inflammation through the degradation of related signaling components, such as TRIF, TRAF, and PELI3 [13–15]. Therefore, TLR4 contributes to its negative feedback regulation through autophagy.

In the current study, to explore the effects of sGRP78 on TLR4’s activity, the behavior of TLR4 in sGRP78-conditioned cells was investigated. Data showed that sGRP78 promoted the formation and internalization of the LPS–TLR4 monomer complex, which was inactive for both the myddosome and triffosome-dependent pathway. After sGRP78 treatment, intracellular TLR4 accumulated to be recognized by p62 for selective autophagy and protein degradation. sGRP78-induced excessive autophagy favored the occurrence of apoptosis and ferroptosis in mouse myeloid cells. These reports establish innovative mechanisms for endotoxin clearance and immune regulation by autophagy-dependent degradation of TLR4, linking innate immunity with multiple ancient processes, including autophagy, apoptosis, and ferroptosis, together through a shared RAMP - sGRP78.

## Method

### Preparation of Recombinant Mouse GRP78

Recombinant mouse GRP78 (rmGRP78) was prepared as described in our previous report [6]. Briefly, a plasmid encoding the full length of mouse GRP78 was transformed into *Escherichia coli* BL21 to generate glutathione-S-transferase (GST)-GRP78. The fusion protein was purified using Pierce® Glutathione Spin Columns (16105; Thermo Scientific, Waltham, MA, USA). rmGRP78 was obtained by thrombin cleavage and identified by SDS–PAGE and immunoblotting. Protein concentration was detected using a Bicinchoninic Acid Protein Assay kit (Beyotime, China). Endotoxins were removed by a pierce high-capacity endotoxin removal resin (88274; Thermo Scientific), and the final endotoxin concentration of protein samples was <10 EU/mg. Negative control (NC) extracts from empty vector-transformed *E. coli* BL21 were prepared in the same way.

### Animals

C57BL/6 mice (HFK Bioscience, Beijing, China), C57BL/6 background TLR4 knockout (KO) mice (kindly donated by Professor Timothy R. Billiar, University of Pittsburgh, Pittsburgh, PA, USA), and CD14 KO mice (The Jackson Laboratory, Bar Harbor, ME, USA) were bred in a specific pathogen-free facility, and female mice were utilized at 6–8 weeks of age.

### Cell culture

RAW264.7 cells were obtained from China Center for Type Culture Collection (Wuhan, China). All cell lines have been authenticated using STR profiling and tested for mycoplasma contamination. RAW264.7 cells were cultured in RPMI 1640 medium containing 10% fetal bovine serum. BMDCs were harvested after 7 days of culture with GM-CSF (20 ng/mL; PeproTech, Rocky Hill, NJ, USA) and IL-4 (10 ng/mL; PeproTech), and BMDMs with M-CSF (50 ng/mL, PeproTech). The purity of BMDCs and BMDMs was more than 90% assessed by flow cytometry. Atg7-knockdown BMDCs (Atg7 KD) were established by siRNA (Ribobio) transfection using riboFECT reagent (Ribobio). LPS (0111:B4, L3024, Sigma-Aldrich), FITC-LPS (0111:B4, F3665, Sigma-Aldrich), z-VAD-fmk (HY-16658B, MedChemExpress), Erastin (E7781, Sigma-Aldrich), Ferrostatin-1 (SML0583, Sigma-Aldrich), Rapamycin (V900930, Sigma-Aldrich), Chloroquine (C6628, Sigma-Aldrich) were supplemented into culture medium respectively in the following experiments.

### Flow cytometry

The following fluorophore-conjugated antibodies were used: PE anti-TLR4 (Biolegend; clone Sa15-21), PE anti-TLR4/MD2 (BD; clone MTS510). Anti-mouse CD16/CD32 (BD; mouse Fc blocker, clone 2.4G2) were used as the blocking reagent to reduce the non-specific binding of the antibodies. Stained cells were read with flow cytometry (LSRII; BD Biosciences, San Jose, CA, USA), and the result was analyzed by FlowJo software (Flow Jo LLC, Ashland, OR, USA).

The efficiency of TLR4 endocytosis was calculated as the ratio of the MFI values of anti-TLR4 (clone Sa15-21) measured from the stimulated cells to those from the unstimulated cells. The extent of TLR4 monomerization was determined by the ratio of the anti-TLR4/MD2 MFI values (clone MTS510) of the stimulated cells to those of the unstimulated cells. For intracellular staining of TLR4/MD2, Cytofix/Cytoperm solution (BD) was added prior to fixation and permeabilization.

### Cell viability assay

1–5 × 10^5^ cells were stained with Annexin V–FITC and propidium iodide (PI) for 15 min at RT in the dark and then analyzed by flow cytometry (LSRII; BD Biosciences, San Jose, CA, USA). The result was analyzed by FlowJo software (FlowJo LLC, Ashland, OR, USA). For LDH release assay, supernatants of sGRP78-conditioned cells (3 × 10^4^) were collected and quantified by measuring released LDH activity using the Cytotoxicity Detection Kit (LDH) according to the manufacturer's instructions (Beyotime, Jiangsu, China). Total iron concentration was measured using Iron Assay Kit (Solarbio, Beijing, China).

### Measurement of ROS production

To detect total reactive oxygen species (ROS), cells were treated with H2DCFDA (10 μM) provided by ROS detection kit (Beyotime, Jiangsu, China) for 30 min in the dark. Control cells were treated with Rosup provided by kit for 30 min as a positive control for increased ROS production. For lipid ROS detection, cells were incubated with C11-BODIPY 581/591 (10 µM) for 30 min. Stained cells were read with flow cytometry.

### DNA ladder

Genomic DNA was extracted from BMDCs using the commercial apoptosis assay kit from Beyotime Company. 10 mg DNA was separated by electrophoresis on 2.5% agarose gels. Separated DNA fragments (DNA ladders) were visualized using a UV transilluminator (Bio-Rad, USA).

### qRT–PCR

Total RNA was extracted using TriZol^®^Reagent (Invitrogen), and cDNA was generated using a HiFiScript^®^ cDNA Synthesis kit (CW Biotech, Beijing, China). qRT-PCR analyses were carried out using an SYBR Green Real-time PCR kit (Toyobo, Osaka, Japan) in a LightCycler^®^ (Bio-Rad Laboratories, Hercules, CA, USA). The expression of individual genes was calculated by a standard curve method and normalized to the expression of GAPDH. Fold changes were analyzed using the formula: 2^−ΔΔCt^.

### Immunoblot

Whole-cell lysates were prepared as previously described [6, 7]. Equal amounts of protein (10–50 μg) were resolved by 12% SDS–PAGE. After electrophoresis, separated proteins were transferred onto nitrocellulose membrane. The membrane was blocked in 5% non-fat milk, followed by overnight incubation with primary antibodies. After incubation with HRP-conjugated secondary antibody, the positive immune reactive signal was detected by ECL (Fude Biotech, Hangzhou, China). Antibodies specific for GST (sc-459, 1:500), CD14 (sc-9150, 1:500), and β-actin (sc-47778, 1:1000) were purchased from Santa Cruz Biotechnology (Santa Cruz, CA, USA). Antibodies specific for GRP78 (ab32618, 1:1000), CD14 (ab182032, 1:1000), and TLR4 (ab13556, 1:1000; ab22048, 1:1000) were obtained from Abcam (Cambridge, UK). Antibodies specific for Atg3 (3415, 1:1000), Atg5 (12994, 1:1000), Atg7 (8558, 1:1000), Atg12 (4180, 1:1000), Atg16L1 (8089, 1:1000) were obtained from Cell Signaling Technology (Danvers, MA, USA). Antibodies specific for Caspase 3 (19677-1-AP, 1:1000), p62 (18420-1-AP, 1:1000) were purchased from Proteintech. Anti-LC3 (PM036, 1:1000) was purchased from MBL life science.

### Native PAGE

Whole-cell lysates were electrophoresed using a 7.5% native PAGE gel (pH 8.8) in a running buffer containing 192 mM glycine, 24 mM Tris, pH 8.3, without detergents, and then transferred onto a PVDF membrane. TLR4 dimers and monomer were detected with a polyclonal rabbit antibody for mouse TLR4 (ab13556, 1:1000) [16]. After incubation with HRP-labeled secondary antibody, the signal was detected by ECL.

### Immunoprecipitation

Cells were lysed in IP lysis buffer (20mM Tris–HCl, pH 7.4, 150mM NaCl, 0.5% Nonidet-P40 and 1× protease inhibitor mixture). The supernatants were mixed with the IP antibody–protein A/G agarose bead (Santa Cruz, USA) and incubated overnight at 4 °C. A small portion of supernatant was saved as input. IP complex was eluted in immunoblot sample buffer and boiled at 95 °C for 5 min. Samples were analyzed by immunoblot as described earlier.

### DNA constructs and transfection

Two plasmids encoding a full length of mouse Tlr4 with His6 epitope (pHIS-TLR4) or FLAG (pFLAG-TLR4) were purchased from Addgene. The Tlr4 were amplified by PCR and then subcloned into pEGFP-N1 (Addgene) to obtain pTLR4-EGFP. BMDCs isolated from *Tlr4*^−/−^ mouse were co-transfected with pHIS-TLR4 and pFLAG-TLR4 for immunoprecipitation assay, or transfected with pTLR4-EGFP for the following cell polarization measurement.

### Fluorescence polarization measurements

pTLR4-EGFP transfected *Tlr4*^−/−^ BMDCs (5 × 10^4^) were pre-treated with sGRP78 (20 μg/mL) and/or LPS (100 ng/mL) for 3h, and then were measured for cell polarization using a Cytation 3 hybrid multi-mode microplate reader (BioTek) equipped with a fluorescence polarization filter (excitation at 430 nm and emission at 480 nm) for 120 min. Polarization was calculated by Gen5 data analysis software (BioTek) according to the following equation:

$${{{\mathrm{mP}}}} = \frac{{{{{I||}}}-{{{G}}} \times {{{I}}} \bot }}{{{{{I||}}} + {{{G}}} \times {{{I}}} \bot }} \times 1000$$

The *G* factor (compensation factor for the plate reader) was set automatically for each measurement based on gain adjustment settings. Polarization change was calculated according to the following equation:

$$\bigtriangleup {{{\mathrm{mP}}}} = {{{\mathrm{mean}}}}\,{{{\mathrm{of}}}}\,{{{\mathrm{mP}}}}\left( {{{{\mathrm{before}}}}\,{{{\mathrm{treatment}}}}} \right) - {{{\mathrm{mP}}}}\left( {{{{\mathrm{after}}}}\,{{{\mathrm{treatment}}}}} \right)$$

### Confocal fluorescent microscopy

After being permeabilized with 0.2% Triton X-100 (Sigma T8787) in PBS, cells were incubated with primary antibodies overnight at 4 °C, then with secondary antibodies and DAPI for 1 h at RT. Cells were mounted with Fluoromount-G (Thermo 00-4958-02) and were imaged with a Zeiss LSM880 confocal microscope. Images were analyzed using ImageJ.

### Transmission electron microscopy

Cells were pre-fixed with 2.5% glutaraldehyde phosphate (0.1 M, pH 7.4) overnight at 4 °C. Post-fixation proceeded in buffered osmium tetroxide, followed by dehydration before embedding in Epon812. Ultrathin sections (80 nm thick) were cut with an ultramicrotome (Leica, EMUC6, Germany), and then stained with uranyl acetate and lead citrate and finally examined by a Tecnai G2 Spirit TWIN transmission electron microscope (FEI, Hillsboro, OR, USA). For each condition, at least 100 cells from randomly chosen fields were observed.

### Statistical analysis

Mean values were compared using the Student's *t*-test (two groups) or one-way ANOVA (three or more groups). Results are the mean and standard error of the mean (SEM). All experiments were performed thrice, and one representative result was presented. The immunofluorescence experiments and protein blots are representative data from at least three independent experiments.

## Results

### sGRP78 promotes endocytosis of LPS–TLR4 complex

First of all, we reconfirmed that sGRP78 treatment could promote TLR4 to be clustered beneath but not evenly distributed at the plasma membrane (Fig. S1A). Using the loss of cell surface expression as a readout for TLR4 endocytosis, we showed that both sGRP78 and LPS induced the rapid TLR4 endocytosis in mouse bone marrow-derived dendritic cells (BMDC) and macrophages (BMDM) (Fig. S1B). However, sGRP78-conditioned cells were defective for LPS-stimulated TLR4 signaling, including TRIF-dependent (IFN-β, CCL5, BST2 expression) and MyD88-dependent pathway (TNF-α, IL-6, IFN-γ expression) (Fig. S1C–F) [6].

**Fig. 1:**
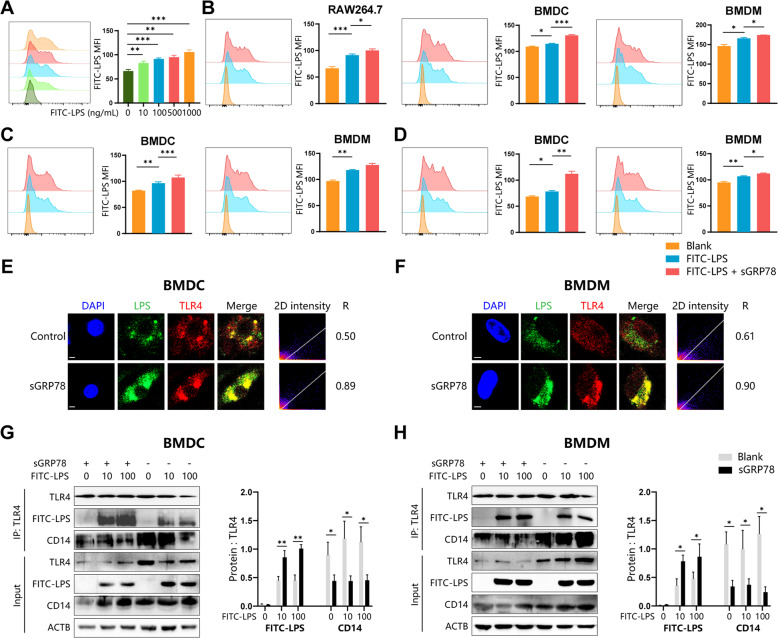
sGRP78 promotes endocytosis of LPS–TLR4 complex. **A** RAW264.7 was treated with FITC-LPS at indicated concentrations for 1 h. **B** Cells were treated with GRP78 (20 μg/mL) and/or FITC-LPS (100 ng/mL) for 1 h. **C**, **D** To remove FITC-LPS fluorescence on the plasma membranes, cells in (**B**) were treated with acid-washing (**C**) or anti-FITC antibodies for fluorescence quenching (**D**). LPS binding was assessed by FCM. Representative histograms (left), and the MFI of FITC-LPS were quantified (right). **E** BMDCs and BMDMs **F** were treated with FITC-LPS for 0.5 h. FITC-LPS and stained TLR4 were detected by confocal microscopy (CLSM). Scale bar = 2 μm. The colocalization between FITC-LPS and TLR4 was described by 2D intensity histogram and Pearson's *R* value. **G**, **H** Immunoprecipitation assay for endogenous TLR4 with FITC-LPS or CD14. Data are representative of three independent experiments with similar results or mean ± SEM from three independent experiments. *P < 0.05, **P < 0.01, and ***P < 0.001.

As a co-receptor, CD14 sensitizes cells to LPS by transferring LPS molecules to TLR4 [17]. sGRP78 was reported to interact with CD14, which might interfere LPS transfer to TLR4 and downstream signaling [6]. To elucidate whether sGRP78 could affect LPS to bind and internalize along with TLR4, LPS binding was tested in sGRP78-conditioned RAW264.7 cells. Data showed that cells bound with FITC-LPS in a dose-dependent manner (Fig. 1A). And sGRP78 treatment increased the MFI of total FITC-LPS (100 ng/mL) in RAW264.7 cells, as well as in BMDCs and BMDMs (Fig. 1B). When using acid washing or anti-FITC antibody to remove or quench FITC-LPS fluorescence on the plasma membranes [16], intracellular FITC-LPS was also increased in sGRP78-conditioned cells (Fig. 1C, D), suggesting that sGRP78 facilitated the internalization of LPS in myeloid cells. Immunofluorescence staining confirmed that GRP78-treated cells endocytosed more FITC-LPS than controls. The intracellular LPS fluorescence co-localized strongly with TLR4 fluorescence with Pearson correlation coefficient as 0.89 in BMDCs and 0.90 in BMDMs (vs. 0.50 and 0.61 in controls, Fig. 1E, F). Moreover, co-immunoprecipitation validated the improved TLR4–LPS interaction but diminished CD14–TLR4 interaction in sGRP78-conditioned cells (Fig. 1G, H). These findings revealed that sGRP78 could promote LPS endocytosis with TLR4.

### sGRP78 enhances TLR4 endocytosis in monomers

**Fig. 2:**
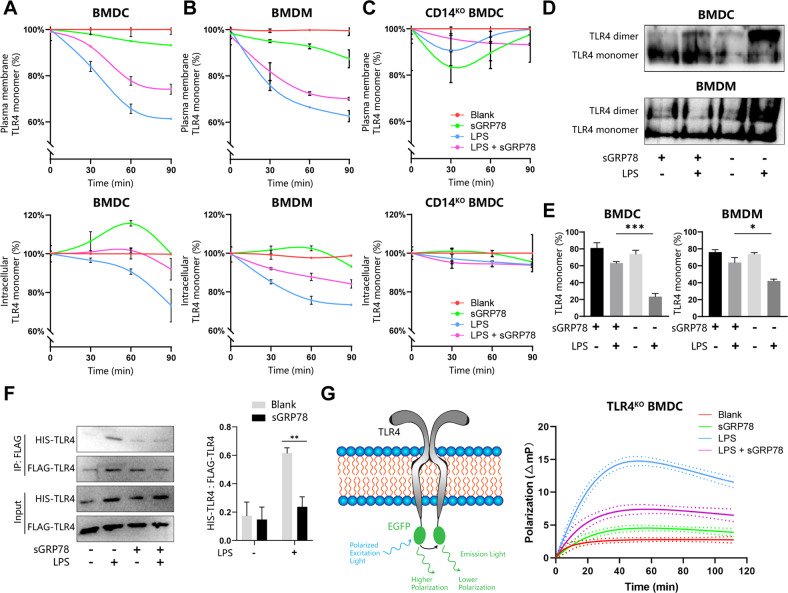
sGRP78 promotes endocytosis of monomeric TLR4 in a CD14-dependent manner. BMDCs (**A**), BMDMs (**B**) and CD14 KO BMDCs (**C**) were treated with sGRP78 and/or LPS (100 ng/mL) for indicated minutes. Representative line chart of plasma membrane (upper) or intracellular (lower) TLR4 monomer were depicted. **D** Native PAGE for detection of the monomeric and dimeric TLR4. **E** Calculation of TLR4 monomer formation using the following formula: TLR4 monomer band/(TLR4 dimer band + TLR4 monomer band). **F** *Tlr4*^−/−^ BMDCs were co-transfected with pHIS-TLR4 and pFLAG-TLR4 for 48 h. Then cells were treated with sGRP78 and/or LPS for 3 h. Cell extracts were immunoprecipitated with anti-FLAG, probed with anti-HIS. **G** Schematic description for the mechanism of detecting monomeric and dimerized TLR4 in TLR4-EGFP transfected *Tlr4*^−/−^ BMDCs (left). LPS and GRP78-dependent polarization responses (right). Higher fluorescence polarization changes (△mP) indicate more monomer-dimer transition. Data are representative of 3 independent experiments with similar results or mean ± SEM from 3 independent experiments. *P < 0.05, **P < 0.01, and ***P < 0.001.

It remains unclear how sGRP78 attenuates the LPS-dependent TLR4 signaling despite its enhancement for LPS–TLR4 interaction and endocytosis (Fig. S1C–F). Tight dimerization of TLR4 is required for the TLR4 downstream signaling pathway [8]. To explore whether sGRP78 treatment could impair this event, TLR4/MD-2 dimerization was assessed using the MTS510 antibody which recognizes monomeric TLR4/MD2 [17]. FCM analysis showed that, consistent with published reports [17], LPS induced the rapid loss of surface and intracellular staining of MTS510 in BMDCs and BMDMs. However, sGRP78 evidently inhibited the LPS-induced TLR4 dimerization and increased intracellular TLR4 monomers by 20% in BMDCs 1 h after sGRP78 treatment (Fig. 2A, B), suggesting sGRP78 prompted monomeric TLR4 endocytosis. In *Cd14*^−/−^ BMDCs, the loss of cytosolic TLR4 monomers staining was almost abrogated (Fig. 2C), confirming the crucial role of CD14 in regulating TLR4 endocytosis and dimerization [17].

The TLR4 monomerization rate was assessed as the ratio between the band intensity of TLR4 monomer and all TLR4 by native PAGE and immunoblot analysis. Data showed that the rate was comparatively low in LPS-treated cells but increased as the supplement of sGRP78 (Fig. 2D, E). In *Tlr4*^−/−^ BMDCs, which were engineered to express HIS-TLR4 and FLAG-TLR4 for evaluating TLR4 oligomerization, immunoprecipitation data manifested that FLAG-TLR4 coprecipitated with more HIS-TLR4 by LPS treatment than by LPS + sGRP78 treatment (Fig. 2F).

Next, we used homo-FRET, which reflects homotypic crowding by altering fluorescence polarization, depending on the proximity relationships between two or more identical fluorophores [18], to dynamically observe TLR4 homotypic interactions inside living cells under sGRP78 treatment. For this purpose, *Tlr4*^−/−^ BMDCs were transiently transfected with pTLR4-EGFP. Dimers in corporating TLR4-EGFP are expected to exhibit lower fluorescence polarization relative to monomer (as shown in Fig. 2G). Results showed that exposure to LPS led to a transient and partially reversible increase in fluorescence polarization change, while the maximal change was about 15 millipolarization (mP) units (Fig. 2G). However, exposure to sGRP78 strongly inhibited the monomer-to-dimer TLR4 transition induced by LPS (Fig. 2G), suggesting sGRP78 could hinder the formulation of TLR4 dimer.

In sum, the above data suggest that sGRP78 inhibits TLR4 dimerization and enhances monomeric TLR4 endocytosis in a CD14-dependent manner in myeloid cells.

### sGRP78 promotes Atg7-dependent autophagy

**Fig. 3:**
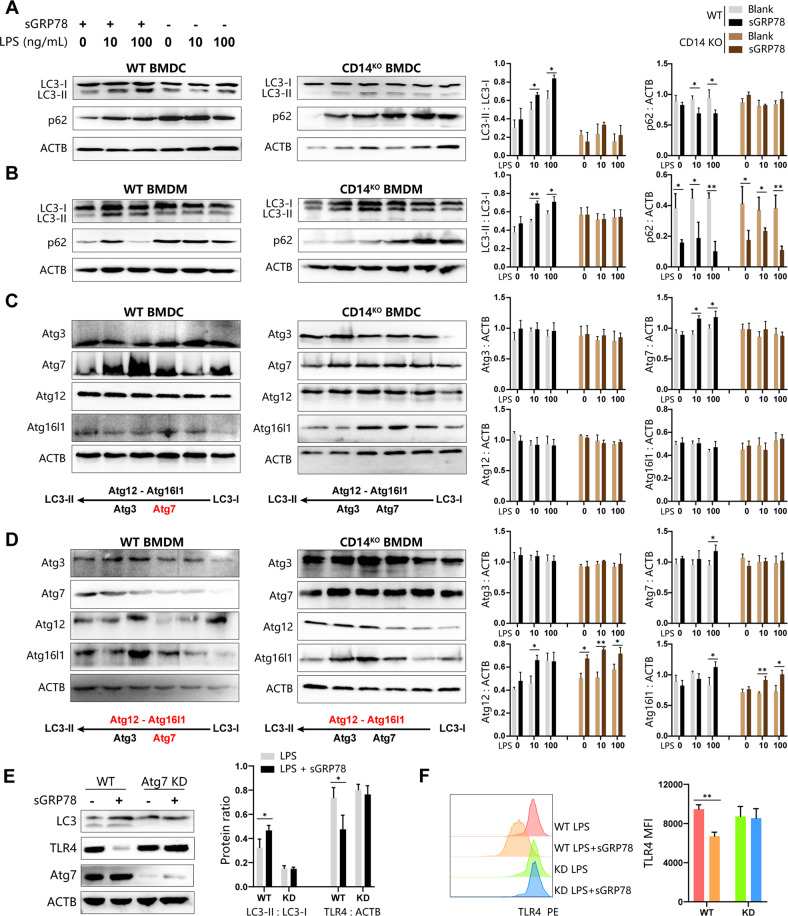
sGRP78 promotes Atg7-dependent autophagy. Cells were treated with sGRP78 (20 μg/ml) and/or LPS (0, 10, 100 ng/ml) for 12 h. Whole-cell lysates from BMDCs (**A**, **C**) or BMDMs (**B**, **D**) were subjected for immunoblot analysis of LC3, p62 (**A**, **B**) and Atg3, Atg7, Atg12, Atg16l1 (**C**, **D**). The upregulated autophagy proteins in GRP78-conditioned cells were highlighted in red in schematic diagram. **E**, **F** LC3, TLR4 and Atg7 expression in Wildtypeand Atg7-knockdown BMDCs. *P < 0.05, **P < 0.01.

We previously reported that after internalization induced by sGRP78, TLR4 would be degraded in lysosomes [6]. However, it remains unclear how TLR4 is trafficked to lysosomes. Srinivasula et al. reported that during TLR4 signaling, protein aggresome was formed and degraded through autophagy [19]. Autophagy is shown to selectively eliminate unwanted, potentially harmful protein aggregates, thereby acting as an effective cytoprotective system [20]. Therefore, it is worth investigating whether sGRP78 would promote the autophagy of protein aggregates to assist in the degradation of TLR4. Our data showed that LPS alone could increase expression of the autophagy marker LC3-II [21] in myeloid cells, while LPS+sGRP78 treatment resulted in a significant increase of LC3-II. Meanwhile, the adaptor protein p62 was evidently degraded in sGRP78-conditioned cells (Fig. 3A, B). *Cd14*^−/−^ cells exhibited unaltered expression and accumulation of LC3-II no matter with/without sGRP78 treatment (Fig. 3A, B). Therefore, sGRP78 promoted the LC3-I conversion to LC3-II in myeloid cells, with CD14 indispensable.

The Atg12–Atg16L1 complex mediates the lipidation reaction during LC3-II formation with the involvement of Atg3 and Atg7, and serves to scaffold the phagophore [20]. Western blot showed that sGRP78 treatment selectively increased Atg7 in BMDCs and Atg7, Atg12, Atg16L1 in BMDMs (Fig. 3C, D), while the Atg12–Atg16L large protein complex was stable in sGRP78-conditioned BMDCs (Fig. 3D). Consistently, sGRP78 failed to induce LC3-II production and TLR4 degradation in Atg7 knockdown BMDCs (Fig. 3E, F). These results suggest that sGRP78-assisted TLR4 degradation through Atg7-dependent autophagy.

### sGRP78 promotes selective autophagy of intracellular TLR4

**Fig. 4:**
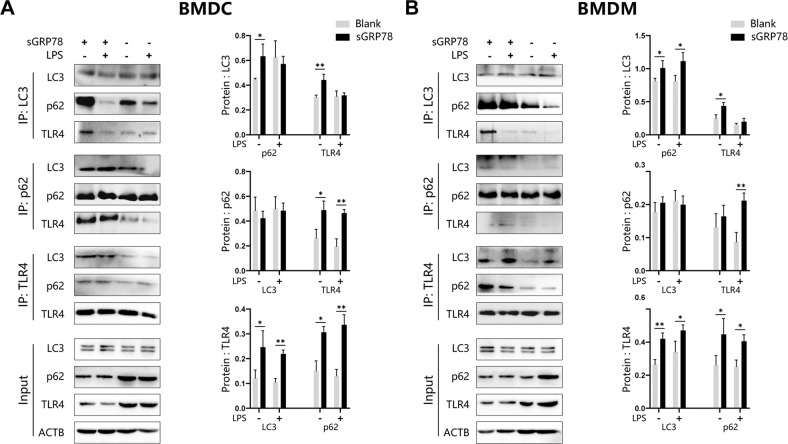
sGRP78 promotes selective autophagy of intracellular TLR4. BMDCs (**A**) and BMDMs (**B**) were treated with sGRP78 and/or LPS for 12 h. Whole-cell lysates were immunoprecipitated with anti-LC3, anti-p62 or anti-TLR4 antibody, respectively. *P < 0.05, **P < 0.01.

Given that sGRP78 interacts with CD14 to enhance monomeric TLR4 endocytosis [6], it is reasonable to speculate that the endocytosed TLR4 monomers might form aggresome to be degraded through autophagy. During cargo sequestration of autophagy, part of the protein aggresome was specifically recognized and removed by autophagy receptors [22, 23]. Immunoprecipitation analysis in Fig. 4 revealed that LPS alone did not stimulate TLR4 to bind with more LC3 and multifunctional autophagy receptor p62, but sGRP78 significantly promoted the binding. And p62-LC3 binding was also remarkably enhanced by sGRP78. Therefore, it was confirmed that sGRP78 facilitated the formation of TLR4–p62–LC3 complexes in myeloid cells, promoting the p62-dependent selection of intracellular TLR4s and LC3-associated autophagosomal membrane expansion.

### sGRP78 promotes the formation of autophagosome

**Fig. 5:**
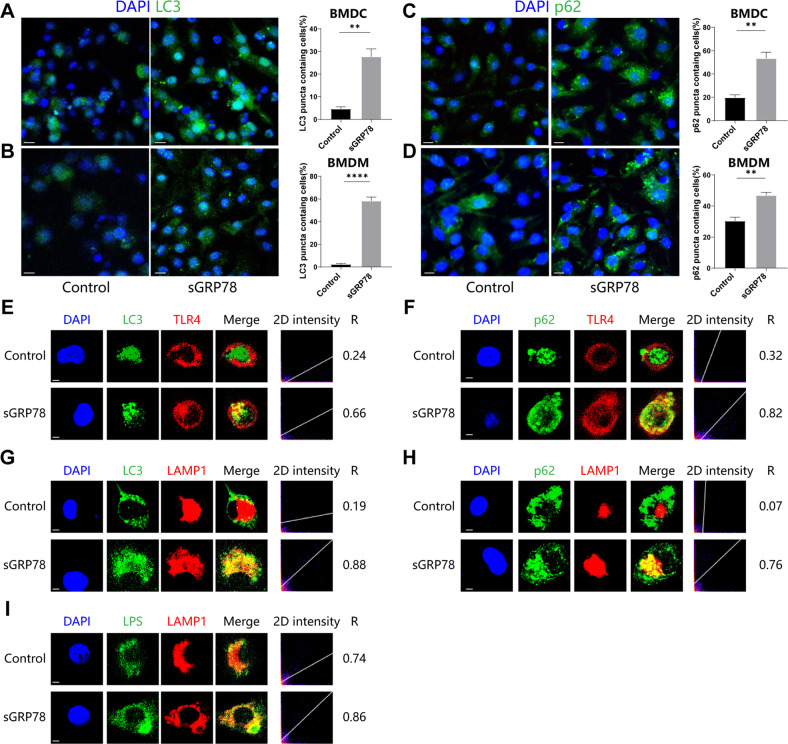
sGRP78 promotes autophagosome formation in myeloid cells. **A**–**D** 12 h after sGRP78 treatment, BMDCs (**A, C**) and BMDMs (**B**, **D**) were immunolabeled with LC3 (**A**, **B**) and p62 (**C**, **D**) antibody. BMDCs (**E**–**H**) or FITC-LPS-treated BMDCs (**I**) were incubated with sGRP78 for 1 h, followed by detection of intracellular LC3, p62, LPS, TLR4, and LAMP1. The colocalization between two markers was described by 2D intensity histogram and Pearson's *R* value. All images are representative of at least three independent experiments in which >100 cells were examined, and >95% of cells showed similar staining. Scale bar = 10 μm (**A**–**D**) or 2 μm (**E**–**H**). **P < 0.01, ****P < 0.0001.

As the last key procedure of autophagy, autophagosomes are formed as the isolation membrane eventually seals into a double-membrane vesicle after cargo sequestration and phagophore expansion [20]. As an autophagosomal marker, LC3 aggregation was evaluated by assessing the formation of GFP-LC3 puncta by fluorescence microscopy [24]. Images showed that sGRP78 treatment resulted in the accumulation of LC3 puncta, as well as p62 puncta, in myeloid cells. BMDMs accumulated more LC3 puncta than BMDCs, implying that autophagosomes are inclined to form in macrophages (Fig. 5A–D). Confocal microscopy analysis further revealed that sGRP78 enhanced colocalization of LC3 or p62 with TLR4 with Pearson's *R* value as 0.66 and 0.82 in BMDCs (Fig. 5E, F), suggesting that sGRP78 enhanced the recruitment of the TLR4–p62–LC3 complex into autophagosomes.

The outer membrane of the autophagosome finally fused with the lysosomal membrane to form an autolysosome, which was followed by the degradation of the autophagic body together with its cargo [20]. Immunofluorescence staining (Fig. 5G–I) revealed an increase in colocalization of LPS, LC3 and p62 with the late endosomal/lysosomal marker protein LAMP-1 in sGRP78-treated cells with Pearson's *R* value as 0.86, 0.88, and 0.78, respectively. Considering our previous report that sGRP78-induced intracellular TLR4 localized with LAMP-1 [6], these results suggested that sGRP78 promoted the fusion of lysosomes with autophagosomes containing LPS–TLR4–p62–LC3 complexes and degraded these complexes in later formed autolysosomes.

### sGRP78 promotes autophagy-dependent apoptosis and ferroptosis of the myeloid cells

**Fig. 6:**
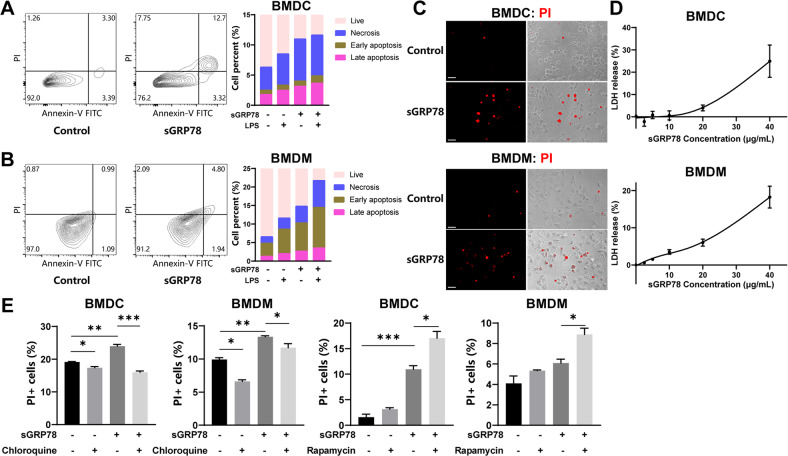
sGRP78 promotes autophagy-dependent death of myeloid cells. BMDCs (**A**) and BMDMs (**B**) were treated with LPS or Grp78 for 24 h followed by AnnexinV/PI staining, **C** PI staining (Scale bar = 10 μm) and **D** LDH release assay. **E** Cells were treated with LPS, Grp78, rapamycin (10 nM), or chloroquine (10 μM) for 24 h. Then cell death was assessed through PI staining by FCM. Data are mean ± SEM from three independent experiments. *P < 0.05, **P < 0.01, and ***P < 0.001.

Although proficient autophagic responses most often operate at the hub of adaptation to stress to mediate cytoprotective effects, excessive autophagy etiologically contributes to cellular demise [25]. In this study, it was observed that sGRP78 led myeloid cells to death, indicated by increased Annexin V and/or PI-positive cell clusters (Fig. 6A, B), PI staining on nuclear (Fig. 6C), and increased LDH release in an sGRP78 dose-dependent manner (Fig. 6D). And compared with sGRP78-secreting 4T1 cells, GRP78-KO 4T1-conditioned BMDCs or BMDMs underwent less death but retained more TLR4 expression on the surface membrane (Fig. S2). Autophagy inhibitor chloroquine significantly decreased the sensitivity of sGRP78 in myeloid cells while autophagy activator rapamycin evidently increased the sensitivity (Fig. 6E). The above data suggest that sGRP78 promotes autophagy-dependent death of the myeloid cells.

**Fig. 7:**
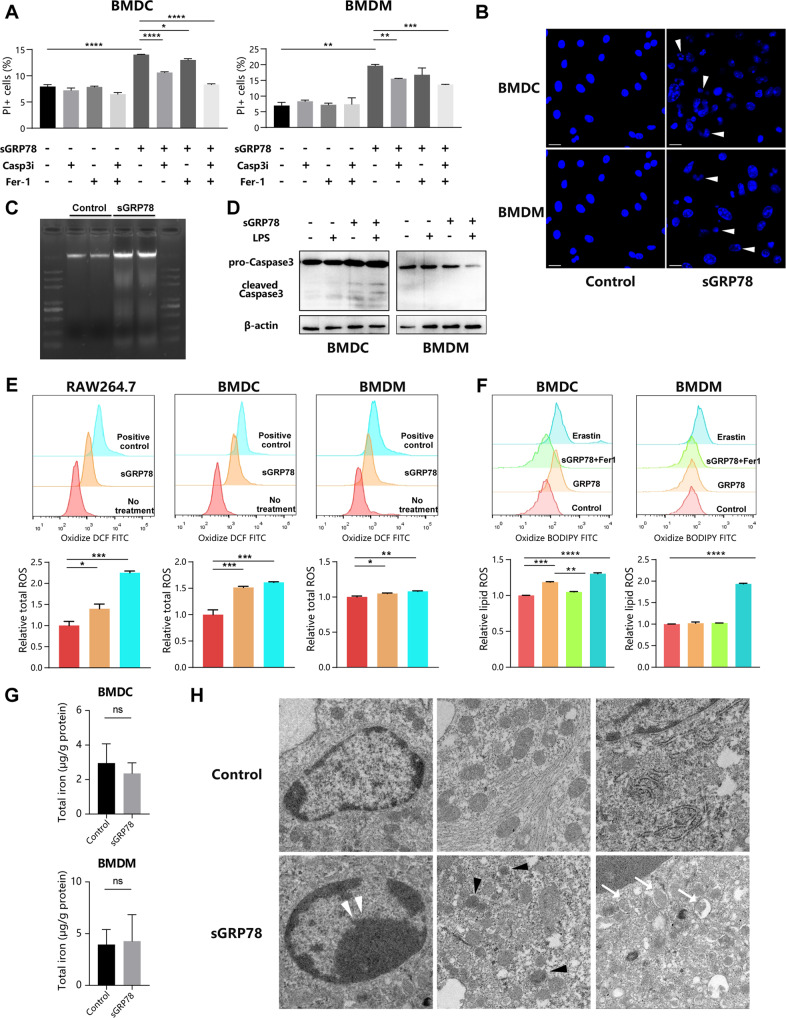
sGRP78 promotes apoptosis and ferroptosis of myeloid cells. **A** Cells were treated with LPS, Grp78, Casp3i (20 μM) and Fer-1 (2 μM) for 24 h. Then cell death was evaluated through PI staining by FCM. Apoptosis of sGRP78-conditioned cells was analyzed by Hoechst 33258 staining (**B**), DNA ladder (**C**) and Caspase 3 cleavage (**D**). **E**, **F** Cells were treated with Grp78, ROS positive control, Fer-1 and Erastin (10 μM). Intracellular and lipid ROS were detected. **G** Total iron concentration. **H** Transmission electron microscopy of BMDCs conditioned with sGRP78 for 48 h. White arrowheads, chromatin condensation; Black arrowheads, shrunken mitochondria; White arrow, the formation of double-membrane vesicles. A minimum of 10 cells per treatment condition was examined. Data are representative of three independent experiments with similar results or mean ± SEM from three independent experiments. *P < 0.05, **P < 0.01, ***P < 0.001, and ****P < 0.0001.

Activated autophagic apparatus can directly cause regulated cell death (RCD), or favors the engagement of other RCD modalities, such as ferroptosis, apoptosis, and necroptosis [25, 26]. When exposed to ferroptosis inhibitors ferrostatin-1 (Fer-1) or caspase 3 inhibitor Ac-DEVD-CHO (Casp3i), sGRP78-treated myeloid cells decreased their death rate (Fig. 7A). Co-administration with Fer-1 and Casp3i inhibited sGRP78-induced death in BMDCs to almost 100%, but only half in BMDMs (Fig. 7A). These findings suggest that sGRP78-induced death in BMDCs happens through apoptosis and ferroptosis, while other unknown mechanisms exist in BMDMs.

In apoptosis assay, other apoptotic features, including chromatin condensation, DNA fragmentation and caspase-3 cleavage (Fig. 7B–D), were also observed in sGRP78-treated BMDCs. In ferroptosis assay, cytosolic ROS was found to be evidently increased in sGRP78-conditioned cells at a parallel level with erastin-stimulated positive control (Fig. 7E). An increased rate of BODIPY oxidation in sGRP78-conditioned BMDCs was found as well. However, this sGRP78-induced lipid hydroperoxides production would be completely inhibited by Fer-1 (Fig. 7F). On the other hand, sGRP78 didn’t influence the total iron concentration in myeloid cells (Fig. 7G), suggesting that sGRP78 promotes ferroptosis by inducing lipid peroxidation without total iron contents influenced. Moreover, sGRP78-conditioned BMDCs exhibited morphological characteristics of autophagy, apoptosis, and ferroptosis, such as the formation of double-membrane enclosed vesicles (autophagy), chromatin condensation and margination (apoptosis), smaller mitochondria with increased membrane density (ferroptosis) (Fig. 7H). In conclusion, sGRP78 prompts apoptosis and ferroptosis in myeloid cells, which is dependent on autophagy.

**Fig. 8:**
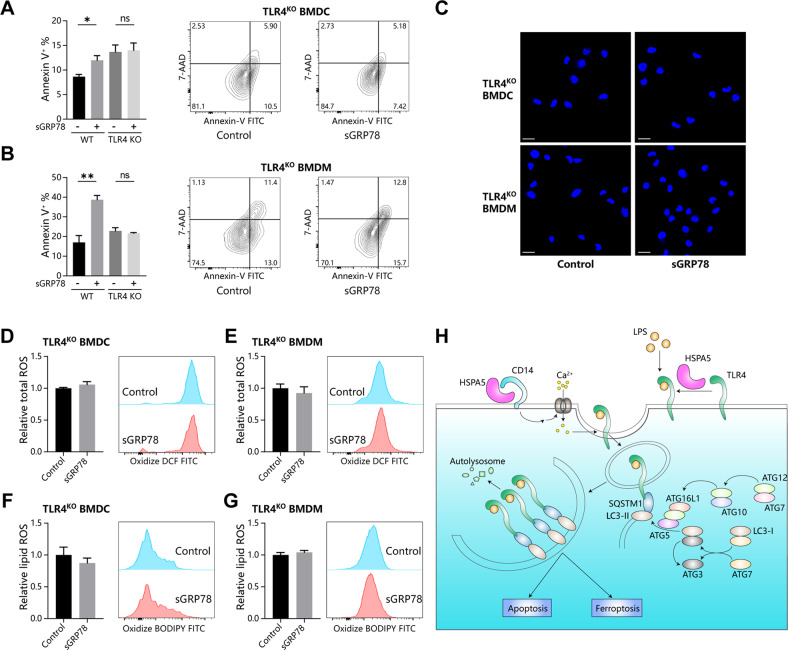
sGRP78-promoted myeloid cells death is TLR4 dependent. BMDCs and BMDMs from WT and TLR4 KO mouse were treated with Grp78 for 24 h. Apoptosis were assayed by AnnexinV/7-AAD staining (**A**, **B**) and by Hoechst 33258 staining (**C**). Scale bar = 10 μm. **D**–**G** Intracellular ROS (**D**, **E**) and lipid ROS (**F**, **G**). Data are mean ± SEM from three independent experiments. *P < 0.05, **P < 0.01. **H** Schematic description for the role of sGRP78 (HSPA5) in inducing selective autophagy of monomeric TLR4 to regulate myeloid cell death.

To clarify the role of TLR4 in sGRP78-induced apoptosis and ferroptosis, we analyzed the effects of sGRP78 on Tlr*4*^−/−^ myeloid cells. It was observed that sGRP78 did not enhance apoptosis of Tlr*4*^−/−^ cells (Fig. 8A–C). In addition, ferroptosis features, such as cytosolic ROS and lipid ROS, were not increased in sGRP78-conditioned Tlr*4*^−/−^ cells (Fig. 8D–G). The above data manifested that sGRP78 had no effects on the apoptosis and ferroptosis in Tlr*4*^−/−^ myeloid cells, suggesting the indispensable role of TLR4 in sGRP78-induced myeloid cell death.

## Discussion

Plenty of studies have suggested the role of GRP78 in inflacommmation resolution in an extracellular form [4–6, 27]. We have reported that one mechanism for sGRP78 to play such a role was through prompting TLR4 degradation at lysosomes [6]. Scientists are investigating how to regulate TLR4 signaling negatively and have described more than multiple regulators of TLR4 until now [8]. However, few regulators directly influence TLR4 itself rather than downstream signaling molecules of TLR4 [28]. Hence, we still lack a clear understanding of how sGRP78 directly functions as an immunomodulator for TLR4-dependent signaling.

In this paper, based on our previous finding that sGRP78 induced the rapid endocytosis of TLR4 with defective TLR4 signaling [6], we further observed that sGRP78 enhanced LPS–TLR4 endocytosis, but the majority of endocytosed TLR4 was in the monomeric form. Upon CD14-mediated transfer of LPS to MD2, monomeric TLR4 dimerizes and then stimulates the assembly of myddosome and triffosomes, which operate in multiple innate immune pathways [8]. Tight dimerization of TLR4 is a prerequisite for signal transduction. The blockade of TLR4 dimerization decreased MyD88 recruitment and TAK1 phosphorylation in Tanshinones-treated RAW264.7 cells [29], definitely attenuating the toxicity of LPS and its signals in globotetraosylceramide-treated primary vascular endothelial cells [30], as well as dramatically reduced IFN-β and pro-inflammatory cytokines in p204-deficient macrophages [31]. In the absence of its co-receptors MD2 and CD14, TLR4 is also maintained in the monomeric form [32]. sGRP78 was reported to mediate endocytosis of TLR4 by targeting CD14 [6]. Hence, its binding with CD14 would cause failed delivery of LPS to TLR4 and could not provoke TLR4 dimerization, keeping TLR4 inactive for downstream signaling pathways in myeloid cells, despite enhanced LPS–TLR4 interaction.

However, not all monomeric TLR4 endocytosis causes defective inflammatory cytokine release. For instance, palmitate directly binds to the monomeric TLR4 complex and triggers its endocytosis in hepatic Kupffer cells, leading to NOX2-mediated ROS generation and IL-1β expression [33]. In order to make it clear how sGRP78 induces defective TLR4 signaling, monomeric TLR4 trafficking was characterized in sGRP78-conditioned myeloid cells. We previously found that after internalization induced by sGRP78, TLR4 is trafficked to lysosomes for degradation [6]. Proteins destined for lysosomal degradation can reach the lysosome through multiple pathways. For instance, Rab7b, a small GTPases, enhanced the degradation and turnover of TLR4 in the lysosomal compartment following LPS stimulation [34]. Moreover, autophagy is one regulated pathway of lysosomal degradation in mammalian cells. GRP78, residing primarily in the endoplasmic reticulum (ER), plays a critical role in facilitating proper protein folding, targeting misfolded protein for proteasome degradation [6]. Arginylated GRP78 is associated with misfolded cytosolic proteins destined for autophagic adaptor p62 (SQSTM1, a ubiquitin-binding protein), allosterically inducing self-oligomerization and aggregation of p62 with LC3 and selective autophagolysosomal co-degradation of GRP78 and p62 together with associated cargoes [35]. In this study, sGRP78 also influenced the selective autophagy of intracellular TLR4 through p62, which bridged between LC3 and ubiquitinated TLR4-p62 [22], finally enhancing the degradation of these complexes in later formed autolysosomes, prompting apoptosis and ferroptosis in myeloid cells.

Autophagy is a fundamental eukaryotic pathway that controls inflammation through regulatory interactions with innate immune signaling pathways by removing inflammasome agonists and affecting immune mediators' secretion [10]. The sGRP78-induced autophagy enhances the delivery of extracellular LPS to the lysosome, enabling the clearance of this PAMP and defective inflammatory cytokines production by DCs and Mφs. And, sGRP78 promoted TLR4, one of the critical PRR in innate immunity, to be selected as the autophagy substrates for degradation. Notably, other fundamental components of triffosomes, including TRIF, TRAF6 were also reported to constitute protein aggresome eliminated by selective autophagy [13, 15]. Thus, we speculate that sGRP78 facilitates the selective autophagy of TLR4-dependent triffosomes to suppress the induction of adaptive immunity.

Autophagy is a multifaceted regulator of cell survival and death through multiple mechanisms. We demonstrated the negative effect of sGRP78-induced autophagy on cell viability. To be termed autophagic cell death, autophagy must mediate death independent of other cell death pathways [26]. However, although autophagy is required, sGRP78 tends to stimulate both apoptosis and ferroptosis in DCs and Mφs. Therefore, we summarized that sGRP78 induced autophagy-dependent cell death rather than autophagic cell death in myeloid cells.

## Conclusion

Our work demonstrated that sGRP78 suppresses TLR4-dependent inflammatory responses through multiple mechanisms (Fig. 8H). Firstly, sGRP78 inhibits TLR4 dimerization to impair TLR4 signaling. Secondly, sGRP78 promoted the endocytosis and selective autophagy of monomeric TLR4 for degradation. Thirdly, sGRP78 induced apoptosis and ferroptosis of myeloid cells to suppress innate immunity. These discoveries suggest that sGRP78 could act as a negative regulator in the innate immune response, suggesting novel means to design TLRs antagonists. In addition, we found an unexpected degree of specificity by which autophagy regulated the turnover of TLR4, which highlights selective autophagy of PRR as a major regulatory pathway in innate immunity.

Overall, our results identified sGRP78 as a typical immunomodulator in TLR4-dependent inflammation with multiple first reported mechanisms and shed new light on the next-generation therapeutics of chronic inflammatory diseases, including sepsis rheumatoid arthritis, asthma, atherosclerosis, and so on.

## Supplementary information

Supplementary figure legend

Supplementray Material

Supplementary Figure 1

Supplementary Figure 2

Reproducibility checklist

## Data Availability

All datasets generated and analysed during this study are included in this published article and its Supplementary Information files. Additional data are available from the corresponding author on reasonable request.
