# Supplementary material for: sGRP78 enhances selective autophagy of monomeric TLR4 to regulate myeloid cell death
Source: Cell Death Dis. 2022 Jul 7;13(7):587. doi: 10.1038/s41419-022-05048-5 (PMC9262968; doi:10.1038/s41419-022-05048-5)
Supplement: Supplementary file 1 — Supplementary figure legend [file 41419_2022_5048_MOESM1_ESM.docx]

**Fig. S1 sGRP78 promotes TLR4 endocytosis and inhibits LPS-induced cytokines release.**

**(A)** BMDCs were treated with AF488 labeled GRP78 at 37°C for 0.5 h. AF488-GRP78 and TLR4 were detected by CLSM. (**B**) Surface TLR4 expression. qRT-PCR **(C-E)** and CBA assay **(F)** for cytokines in RAW264.7 (**C**), BMDCs (**D**) and BMDMs (**E**). Error bars represent mean ± SEM from one of three representative experiments. **P* < 0.05, ***P* < 0.01, and ****P* < 0.001.

**Fig. S2 sGRP78 ablation upregulates TLR4 expression and inhibits death of myeloid cells**

**(A)** sGRP78 released by different cell lines. **(B)** BMDCs and BMDMs were co-cultured with WT or GRP78-knockout 4T1 cells through a transwell. **(C)** TLR4 expressions. **(D)** Apoptosis of myeloid cells.
