# Supplementary material for: sGRP78 enhances selective autophagy of monomeric TLR4 to regulate myeloid cell death
Source: Cell Death Dis. 2022 Jul 7;13(7):587. doi: 10.1038/s41419-022-05048-5 (PMC9262968; doi:10.1038/s41419-022-05048-5)

1G

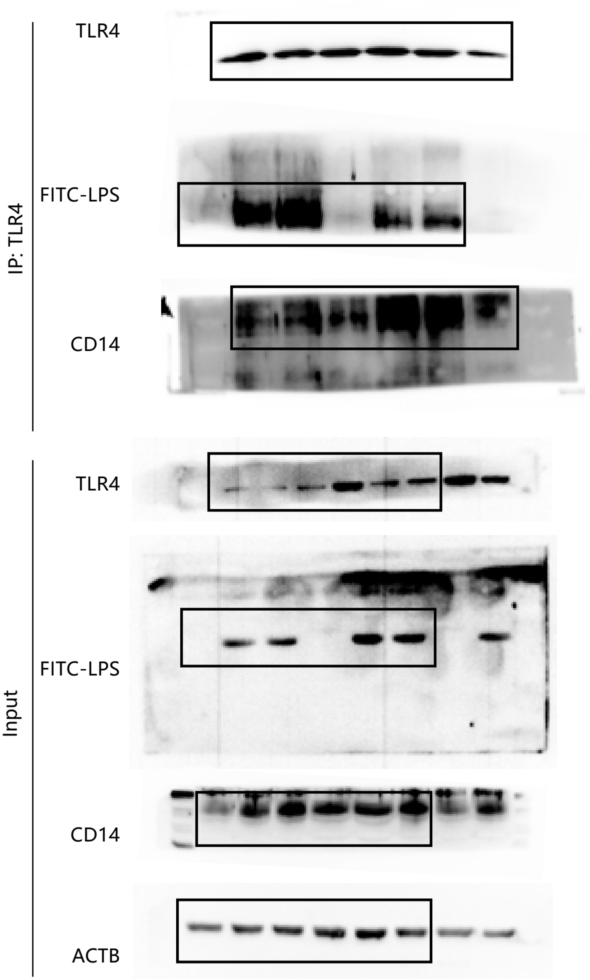
IP: TLR4
TLR4
FITC-LPS
CD14
Input
TLR4
FITC-LPS
CD14
ACTB

1H

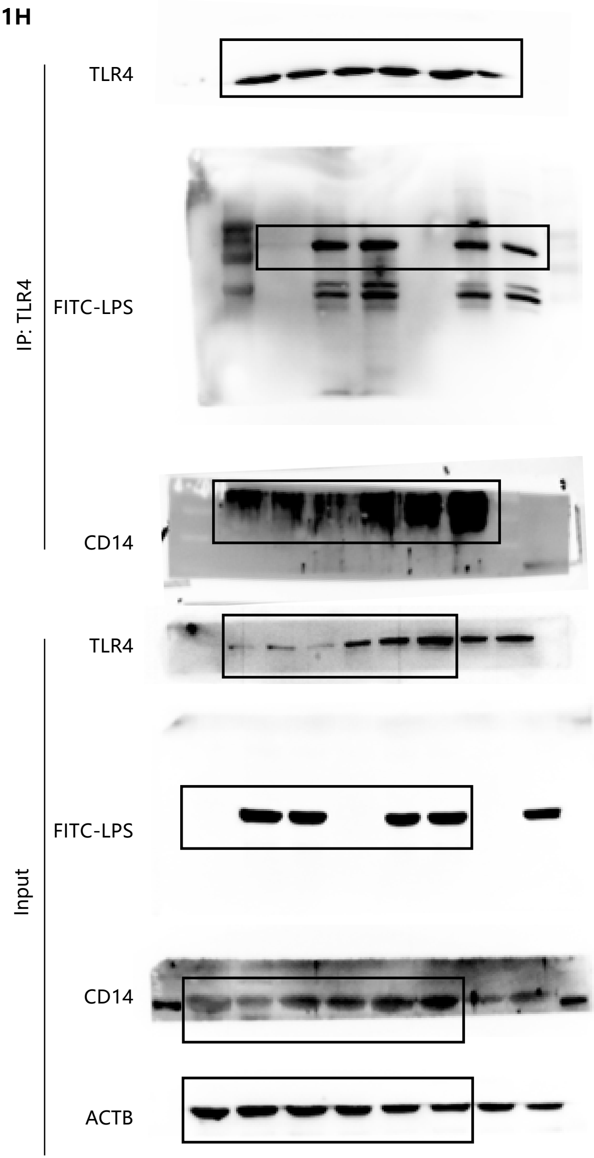
IP: TLR4
TLR4
FITC-LPS
CD14
Input
TLR4
FITC-LPS
CD14
ACTB

2D

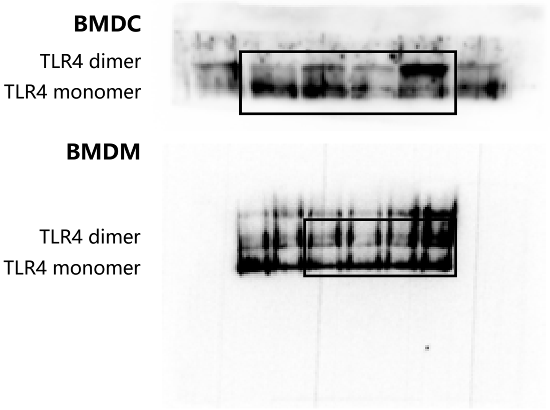
BMDC
TLR4 dimer
TLR4 monomer
BMDM
TLR4 dimer
TLR4 monomer

2F

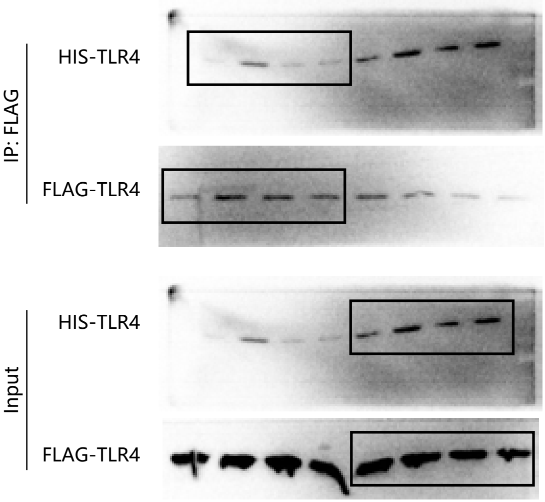
IP: FLAG
HIS-TLR4
FLAG-TLR4
Input
HIS-TLR4
FLAG-TLR4

3A

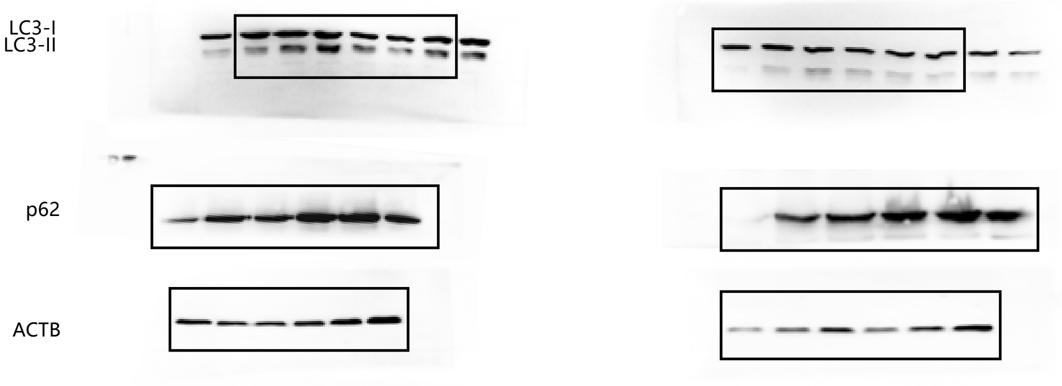
LC3-I
LC3-II
p62
ACTB

3B

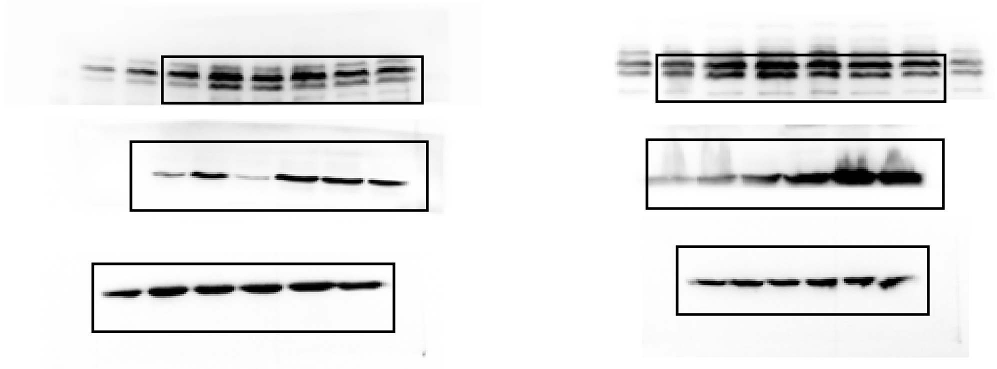

3C

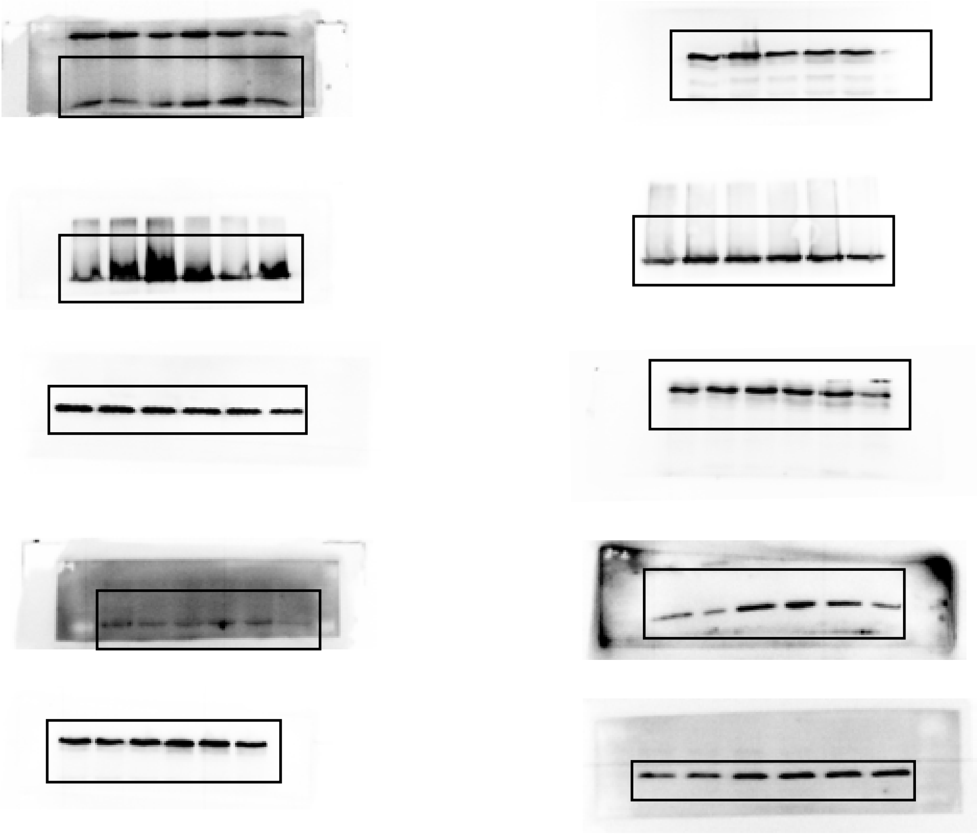

3D

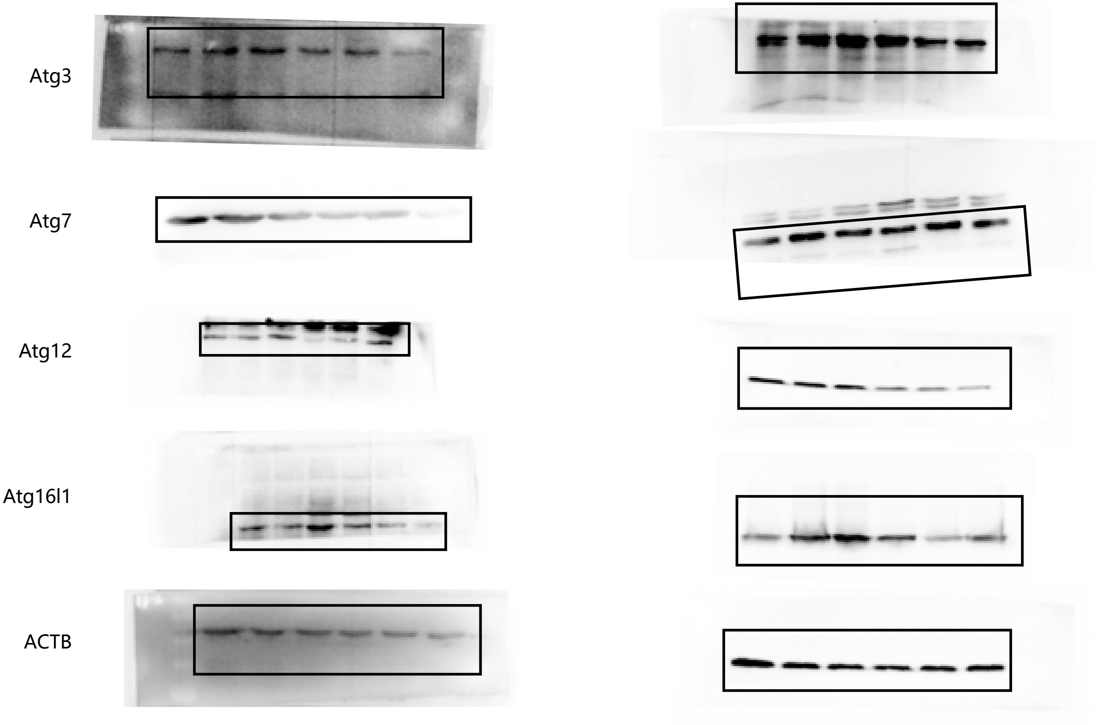

3E

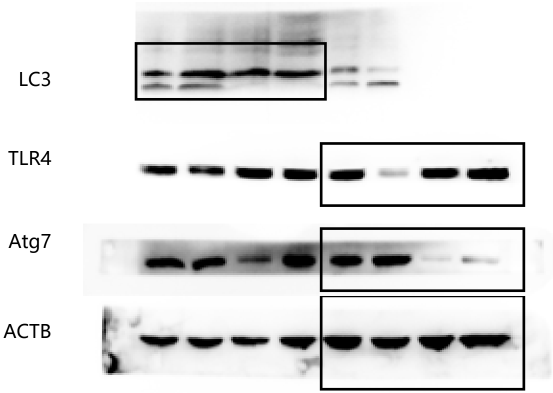

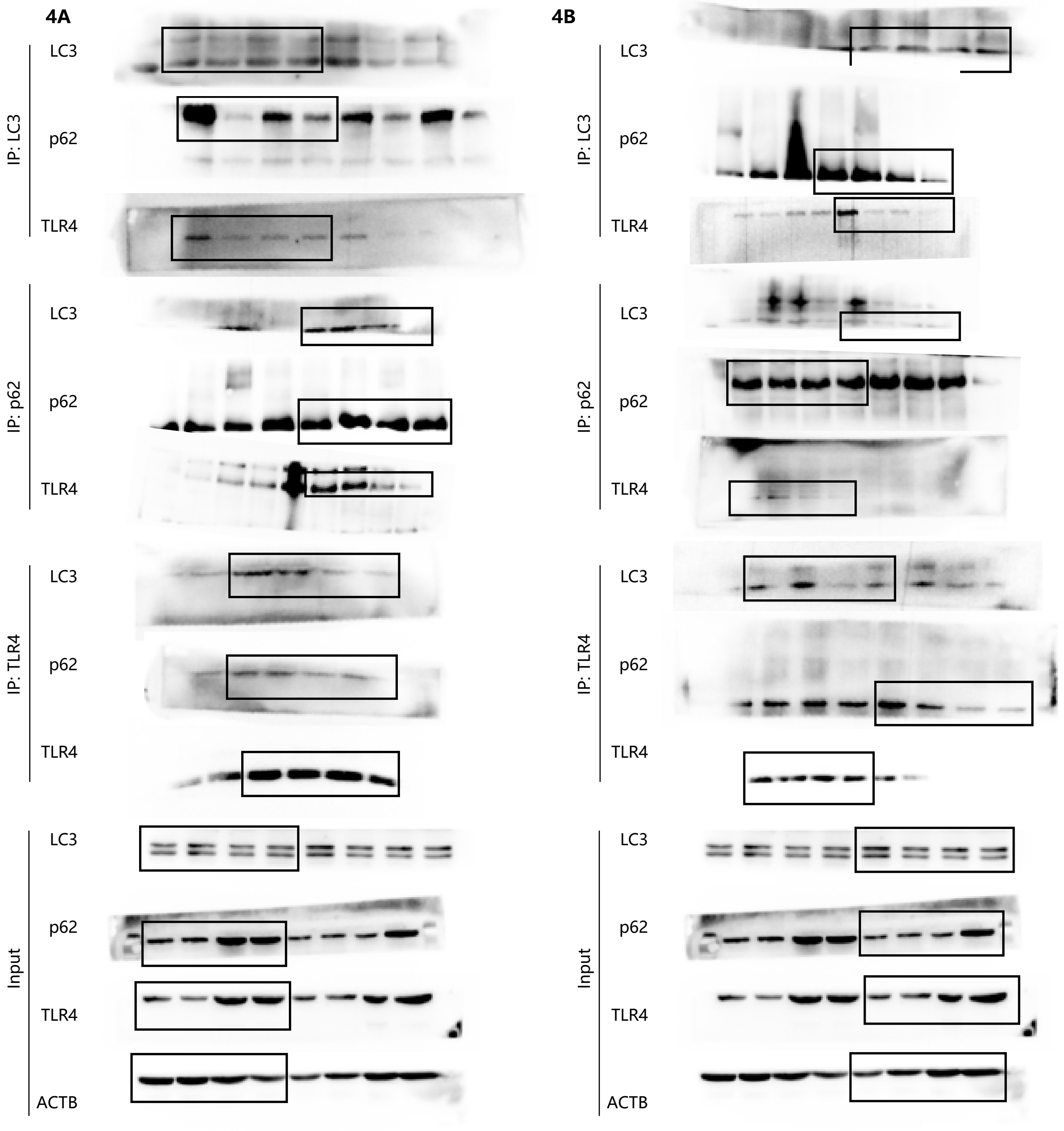
4A
IP: LC3
LC3
p62
TLR4
IP: p62
LC3
p62
TLR4
IP: TLR4
LC3
p62
TLR4
Input
LC3
p62
TLR4
ACTB
4B
IP: LC3
LC3
p62
TLR4
IP: p62
LC3
p62
TLR4
IP: TLR4
LC3
p62
TLR4
Input
LC3
p62
TLR4
ACTB

7D

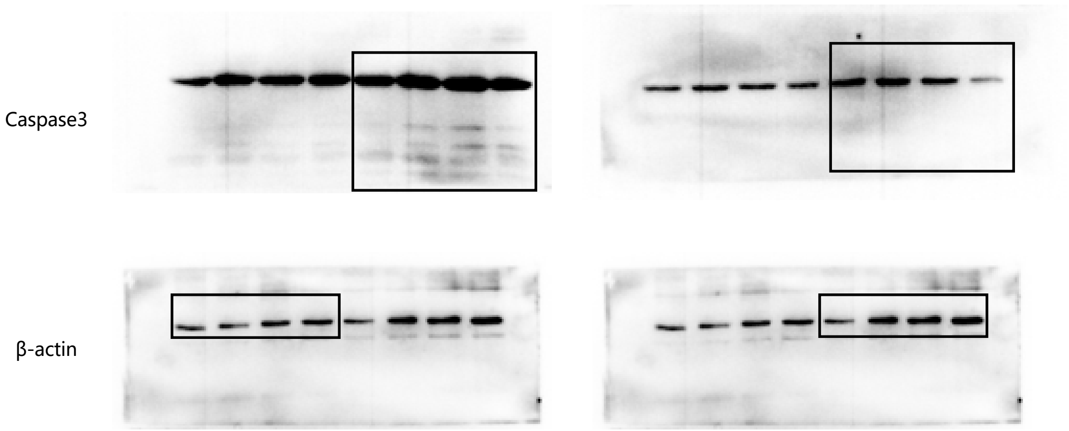

Supplement: Supplementary file 2 — Supplementray Material [file 41419_2022_5048_MOESM2_ESM.pdf]
