# Supplementary figures and images for: sGRP78 enhances selective autophagy of monomeric TLR4 to regulate myeloid cell death
Source: Cell Death Dis. 2022 Jul 7;13(7):587. doi: 10.1038/s41419-022-05048-5 (PMC9262968; doi:10.1038/s41419-022-05048-5)

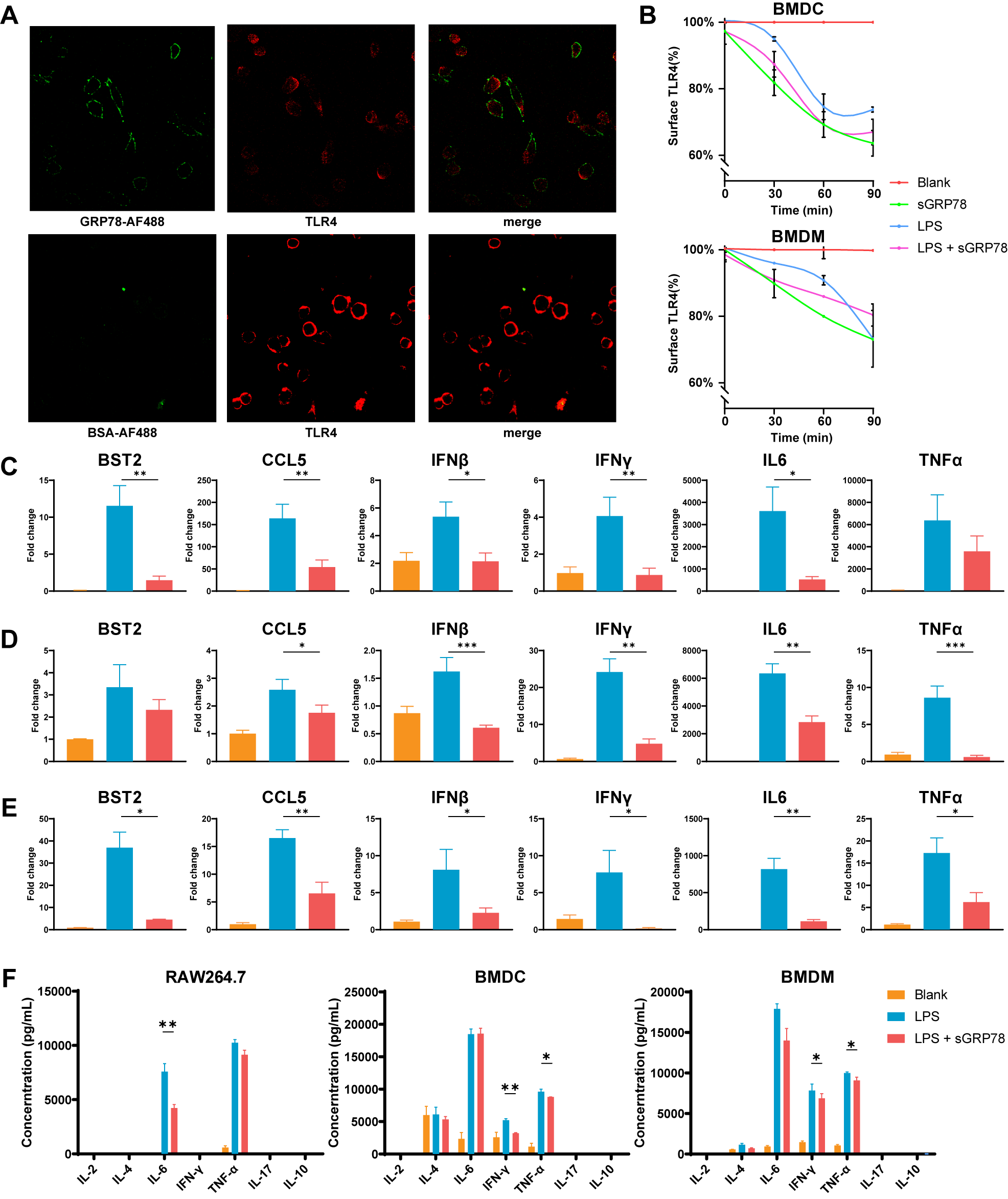

Supplement: Supplementary file 3 — Supplementary Figure 1 [file 41419_2022_5048_MOESM3_ESM.tif]

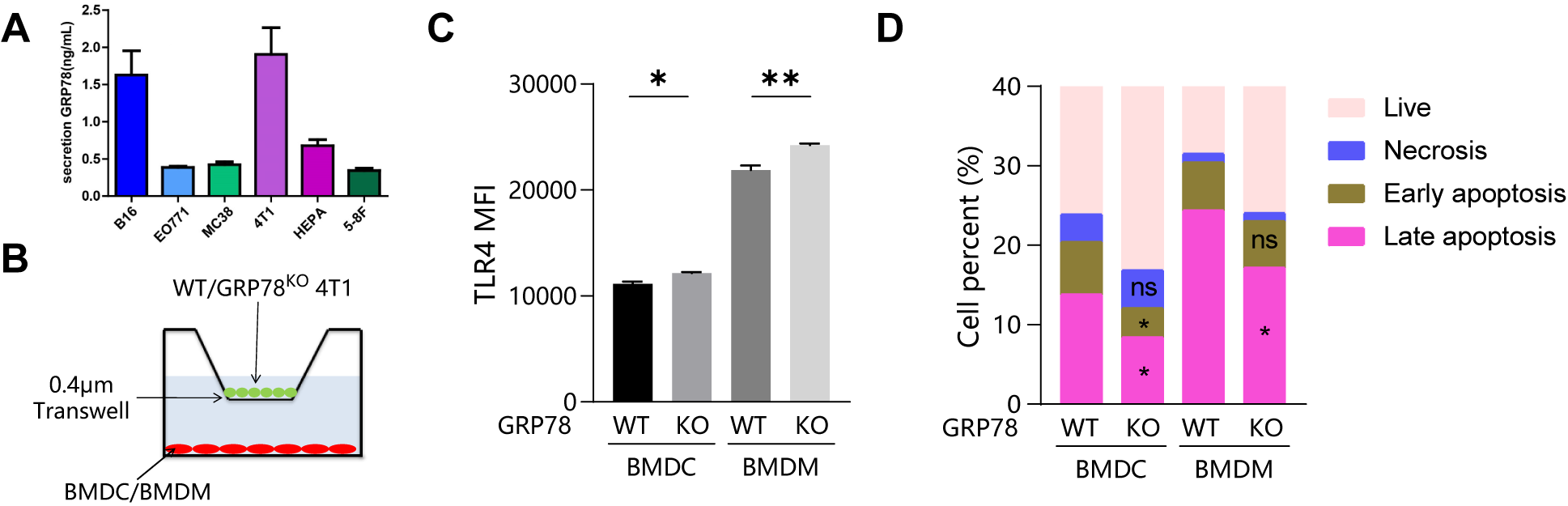

Supplement: Supplementary file 4 — Supplementary Figure 2 [file 41419_2022_5048_MOESM4_ESM.tif]
